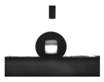# Dopamine-Dyed and Functionally Finished Silk with Rapid Oxidation Polymerization

**DOI:** 10.3390/polym10070728

**Published:** 2018-07-03

**Authors:** Biaobiao Yan, Qingqing Zhou, Tieling Xing, Guoqiang Chen

**Affiliations:** National Engineering Laboratory for Modern Silk, Soochow University, Suzhou 215123, China; 20175215061@stu.suda.edu.cn (B.Y.); zhouqingqing716@163.com (Q.Z.); chenguojiang@suda.edu.cn (G.C.)

**Keywords:** silk fabric, dopamine, rapid oxidation polymerization, dyeing, anti-UV, hydrophobicity

## Abstract

Nowadays, more and more attention has been paid to ecological environment problems, and the dyeing and finishing field is no exception. Environmentally friendly dyeing and finishing methods have been extensively studied. Inspired by the bioadhesive force of marine mussels, dopamine (DA) was applied as a dyestuff and investigated in textile dyeing. In this work, dopamine was dyed on silk with a rapid oxidation polymerization in the presence of metal ions (Fe^3+^) and sodium perborate oxidant (Ox). The polydopamine (PDA) was rapidly deposited on silk fabric and the dyeing process was optimized as follows: the concentration of DA was 2 g·L^−1^, and that of Fe^3+^ was 2 mmol·L^−1^; the total reaction time was 50 min and reacted at 50 °C; 9 mmol·L^−1^ Ox was added at 20 min. The K/S value of the treated silk fabric reached 11.46. The color fastness of dyed fabric to light fastness reached Level 4. The SEM and AFM tests showed that the particles attached to the fabric surface and increased the roughness. The XPS test further proved that polydopamine (PDA) was deposited on the fabric. The treated fabric also had a good anti-UV property with a UPF >30 and UVA <4%. The water contact angle of treated fabric attained 142.6°, showing better hydrophobicity, and the weft breaking strength was also improved. This environmentally friendly dyeing and finishing method can be applied and extended to other fabrics.

## 1. Introduction

In recent years, the problem of environmental pollution has aroused widespread concern [1]. The textile industry has become the focus of public opinion because it discharges a large amount of wastewater and pollutes water resources [2,3]. Ecological dyeing technology is popular because of its “green” nature. Short flow [4], anhydrous dyeing [5], and low temperature dyeing [6] techniques have been studied, but there is still a long way to go before large-scale production can be achieved.

Since ancient times, natural dyes have been used in daily necessities because of their simplicity, convenience, and ease of use [7]. In modern times, synthetic dyes have gradually occupied the dye market for cheap and convenient production. However, some synthetic dye precursors or products that are carcinogenic to humans are banned [8]. Natural dyes are considered nontoxic and environmentally friendly. However, natural dyes have obvious defects. Natural dyes have low dye uptake on fibers, and the dyed fabrics have poor color fastness, which limits their application [9].

Dopamine (DA) is derived from chemicals secreted by marine mussels, because it has excellent adhesion and no selectivity to the substrates, and can be used for material modification. Since Lee [10] used dopamine for the first time as a material modifier, DA has been widely studied and successfully used for preparation of functional materials with excellent performance. These functional materials with superhydrophobic and oil–water separation [11,12,13,14], antibacterial [15,16], UV protection [17,18], and dye adsorption [19,20] properties could be successfully prepared by grafting other chemical components onto the polydopamine (PDA)-coated material surface through the excellent adhesion force.

Since Xin [21] found that PDA can dye the surface of the material, and Jeon [22] added different metal ions in the PDA solution to hair and obtained hair products of different colors. Some scholars have attempted to apply PDA in textile dyeing and functional finishing. Jia’s team [23] found that laccase can catalyze the polymerization of DA, and more PDA can be coated on silk fabric surface under certain conditions. Mao [24] modified the fabric surface through electroless silver plating to prepare polyester fabric with good conductivity. Yuan [25] utilized DA’s resistance to acids and bases to improve the corrosion resistance of fabric. These methods are unique and ideal, but they all require a long time to obtain a large amount of PDA under alkaline conditions [26], which affects the application of DA in the textile field.

Studies have found that the polymerization rate of DA is too slow and usually takes more than 10 h or even several days [27,28]. For this reason, methods have been adopted, such as ultraviolet radiation [29], electrochemical acceleration [30], and oxidation catalysis [31], to accelerate the reaction, but the effect is minimal. It is important to find a rapid method of polymerizing DA on the surface of materials [32,33].

In this work, oxidant sodium perborate was used to accelerate the polymerization of DA, and non-toxic Fe^3+^ was chosen as the catalyst of oxidation polymerization and as the bridge between DA and fibers. The effect of PDA dyeing was evaluated by the K/S value of silk fabric. The effects of adding time of Ox, Ox concentration, Fe^3+^ concentration, and total reaction time on the PDA-dyed fabric were quantitatively analyzed. Scanning electron microscopy (SEM), atomic force microscopy (AFM), and X-ray electron spectroscopy (XPS) were used to characterize the surface of the dyed fabric. The mechanical strength, UV resistance, and water contact angle of the dyed fabric were also measured. Finally, a short process of dyeing and functional finishing of silk fabric is put forward.

## 2. Materials and Methods

### 2.1. Materials and Reagents

The degummed silk fabric, with a 36 g/m^2^ density, was purchased from Huajia Silk Group Co. Ltd., Suzhou, China. Dopamine hydrochloride (98.5% purity) was supplied by Yuanye Biotechnology Co., Ltd., Shanghai, China. Ferric chloride was purchased from Sinopharm Chemical Reagent Co., Ltd., Shanghai, China. Sodium perborate tetrahydrate was purchased from Shanghai Titan Scientific Co., Ltd., Shanghai, China.

### 2.2. The Preparation of PDA–Fe^3+^–Silk Fabric

**Figure 1:**
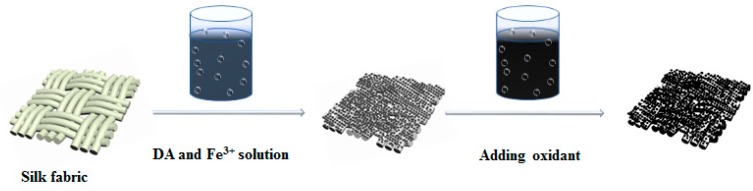
Schematic of dopamine (DA) silk dyeing flow process.

PDA–Fe^3+^–silk fabric was performed by the “dip-reduced-dry” method (Figure 1). The different silk fabric samples were treated according to the following steps, respectively: DA treatment (DA): The degummed silk fabric was immersed into DA solution (2 g·L^−1^) for 50 min at 50 °C. DA and Fe^3+^ treatment (DA–Fe^3+^): the degummed silk fabric was immersed with DA solution (2 g·L^−1^) and Fe^3+^ solutions for 50 min at 50 °C. DA and Ox (sodium perborate tetrahydrate) treatment (DA–Ox): the degummed silk fabrics was immersed with DA solution (2 g·L^−1^) for 50 min at 50 °C. It must be noted that the Ox was added to the solution after 20 min of reaction. DA, Fe^3+^, and Ox treatment (DA–Fe^3+^–Ox): the degummed silk fabric was immersed with DA solutions (2 g·L^−1^) and Fe^3+^ solutions for 50 min at 50 °C, and the Ox was added to the solution after 20 min of reaction. The treated fabrics were washed with deionized water and dried in a vacuum condition.

### 2.3. Characterization and Measurements

#### 2.3.1. Color Measurement

Color characteristic values (L*, a*, b*, c*, *h*) and *K*/*S* values of the dyed fabric were tested using a Hunter Lab Ultra Scan PRO reflectance spectrophotometer (illuminant D65; 10° standard observer, (Hitachi High Technologies America, Inc., Schaumburg, IL, USA). The range of wavelength was 350–750 nm.

#### 2.3.2. The Color Fastness Test

The color fastness of dyed silk fabric was evaluated according to GB/T 3921-2008: color fastness to washing with soap, GB/T 3920-2008: color fastness to rubbing and GB/T 8426-1998: light color fastness: daylight.

#### 2.3.3. The Tensile Fracture Strength Test

The INSTRON-3365 material testing machine (American INSTRON Company, Norwood, MA, USA) was used to test the strength of fabric. The sample size was 30 cm × 5 cm, and the clamping length was 20 cm. Under the INSTRON-3365 material testing machine, the warp and weft breaking strength of the fabric were tested, and the stretching speed was 10 cm/min.

#### 2.3.4. SEM Analysis

The Hitachi TM 3030 desktop scanning electron microscope (Hitachi Ltd., Tokyo, Japan) was used to observe the surface morphology of silk fibers and the test voltage was maintained at 15 kV.

#### 2.3.5. AFM Analysis

The Multiomode 8 atomic force microscope (Bruker, Billerica, MA, USA) was used to test the surface morphology of fabric samples, and the measurement range was 12 µm × 12 µm in the X and Y directions, and 2.5 µm in the Z direction.

#### 2.3.6. XPS Study

The surface element content of silk samples was analyzed by Thermo ESCALAB 250XI X-ray photoelectron spectroscopy (Thermo Fisher Scientific Co., Ltd., Waltham, MA, USA) using an Al Ka X-ray source (1484.6 eV). 

#### 2.3.7. The UV-Protection Factor of Colored Silk Fabric

The ultraviolet protection factor (UPF) of silk fabric was measured with the Lab Sphere UV-1000F Transmittance Analyzer (Lab sphere, Inc., Northsutton, VA, USA). The measurement result was the average of four tests on a single layer of fabric.

#### 2.3.8. Water Contact Angle Measurement

The KRUSS DSA 100 (Kruss, Filderstadt, Germany) was used to evaluate the water contact angle of the fabric.

## 3. Results and Discussion

### 3.1. Effect of Reaction Conditions on the Dyeing of PDA Silk

**Figure 2:**
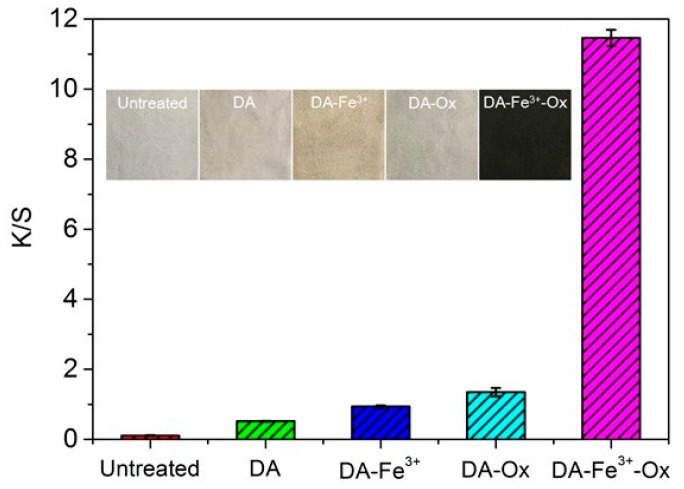
Effect of adding different substances on *K*/*S* of DA-dyed silk. (DA = 2 g·L^−1^, Fe^3+^ = 2 mmol·L^−1^, Ox = 9 mmol·L^−1^, at 50 min and 50 °C).

The color depth of dyed silk sample was evaluated via *K*/*S* values. Figure 2 and Table 1 show the changes in the *K*/*S* and L* a* b* values of the samples treated with different substances.

It is clear from Figure 2 that there is a great difference in the color of silk fabric with the addition of different substances because of the various amount of PDA on the fabric surface. There was a small amount of PDA on the surface of the fabric treated with DA for 50 min without other reagents. For samples treated with DA–Fe^3+^ or DA–Ox separately, it can be found that the color depth increased to a certain extent. However, the color depth was still quite low. Further, it was found that more DA could be polymerized when Fe^3+^ and Ox were added together under suitable conditions, and the fabric was dyed in deep black. The color characteristic value of silk samples are listed in Table 2. L* represents the lightness of fabric, and the minimum for L* is zero, which means black; positive a* is red, and negative a* is green; positive b* is yellow, and negative b* is blue; c* and *h* reflect the fabric brightness and hue. In general, the a* and b* values, brightness, and hue of silk fabric treated by DA–Fe^3+^–Ox greatly changed compared with the untreated and other treated samples. The L* value of silk fabric treated by DA–Fe^3+^–Ox was the smallest, indicating that silk was dyed most deeply in this way. 

It is well known that Fe^3+^ can induce polyphenols to form complex compounds [34] and can be used as a bridge to combine DA with the silk fabric. On the one hand, DA can also slowly oxidatively polymerize in air, and the addition of Ox can accelerate the polymerization of DA. On the other hand, the combination of Fe^3+^ and DA also endows a certain color with silk fabric. When silk fabric was treated with DA–Fe^3+^–Ox, Fe^3+^ can function as a catalyst of Ox and greatly accelerate the rapid oxidation polymerization of DA. Compared with the DA–Fe^3+^ and DA–Ox samples with a very low *K*/*S* value, the *K*/*S* value of DA–Fe^3+^–Ox sample was huge increased. This phenomenon indicates that the combination of Fe^3+^ and Ox is effective, and the addition of Fe^3+^ and Ox will be applied in the following experiments.

**Figure 3:**
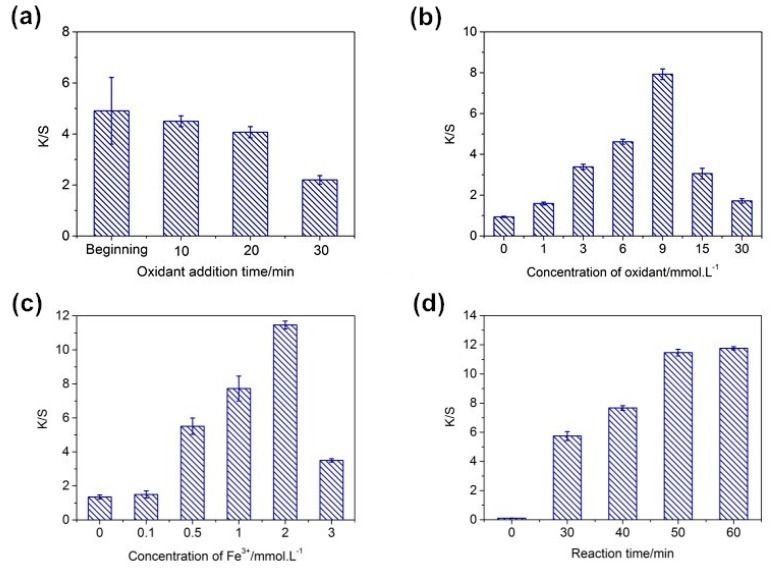
The effect of time of Ox addition (**a**), Ox concentration (**b**), Fe^3+^ concentration (**c**), and reaction time (**d**) on the *K*/*S* values of silk fabric. ((**a**) DA = 2 g·L^−1^, Fe^3+^ = 1 mmol·L^−1^, Ox = 15 mmol·L^−1^, *T* = 50 °C, *t* = 40 min; (**b**) DA = 2 g·L^−1^, Fe^3+^ = 1 mmol·L^−1^, *T* = 50 °C, *t* = 40 min (adding Ox at 20 min); (**c**) DA = 2 g·L^−1^, Ox = 9 mmol·L^−1^, *T* = 50 °C, *t* = 40 min (adding Ox at 20 min); (**d**) DA = 2 g·L^−1^, Fe^3+^ = 2 mmol·L^−1^, Ox = 9 mmol·L^−1^, *T* = 50 °C (adding Ox at 20 min)).

It was also found that the time of adding Ox had a great influence on the results. Accordingly, Ox was added in the reaction solution at the beginning and at 10, 20, and 30 min of reaction time to explore the reasonable addition time. Figure 3a shows that the *K*/*S* value of the dyed fabric greatly increased at the beginning of the reaction, but the color depth of the fabric was very uneven and the deviation was quite large. The reason was that the earlier addition of Ox led to the formation of more PDA, resulting in an uneven distribution of PDA on silk fabric. Consequently, the appropriate Ox addition time was chosen to be 20 min.

The concentration of the Ox and the added Fe^3+^ also affected the *K*/*S* value of the dyed fabric. With their concentration increasing, the *K*/*S* value increased first and then decreased. A higher concentration of Ox and Fe^3+^ promoted the polymerization of DA, resulting in more PDA deposition on the fabric. However, this effect was weakened when the amount of Ox and Fe^3+^ reached a certain concentration, which was reflected by the decrease in the color depth of silk fabric at higher concentrations of Ox and Fe^3+^. This could be explained by the fact that a greater amount of PDA formed in the solution was unfavorable for them to be absorbed by the silk surface. Therefore, the optimum dosages of Ox and Fe^3+^ were 9 mmol·L^−1^ and 2 mmol·L^−1^, respectively.

As a potential dyestuff used for industrial production, the reaction time of DA-dyed fabric is critical. Most current reports on the coloration of PDA materials require an overnight reaction, which limits its application. Under the appropriate conditions, the optimal reaction time was discussed. Figure 3d shows that, with the increase in reaction time, the color depth of fabric gradually increased, indicating that the deposition amount of PDA on the fabric was large and that 50 min of dyed fabric can reach a higher *K*/*S* value.

### 3.2. The Color Fastness Test

Table 2 shows the color fastness of dyed silk after DA treatment. The results indicated that, for the sample treated with DA and using Fe^3+^ as a catalyst, followed by Ox acceleration, silk with deep black color could be obtained. Except for the wet rubbing fastness, which reached Level 3 or 4, the washing fastness, dry rubbing fastness, and light fastness all reached Level 4, indicating that the adhesion of the PDA from marine mussels was quite high. In addition to the textile field, it is expected that the almost perfect bonding force of DA will expand its application range.

### 3.3. The Fabric Strength Test

**Figure 4:**
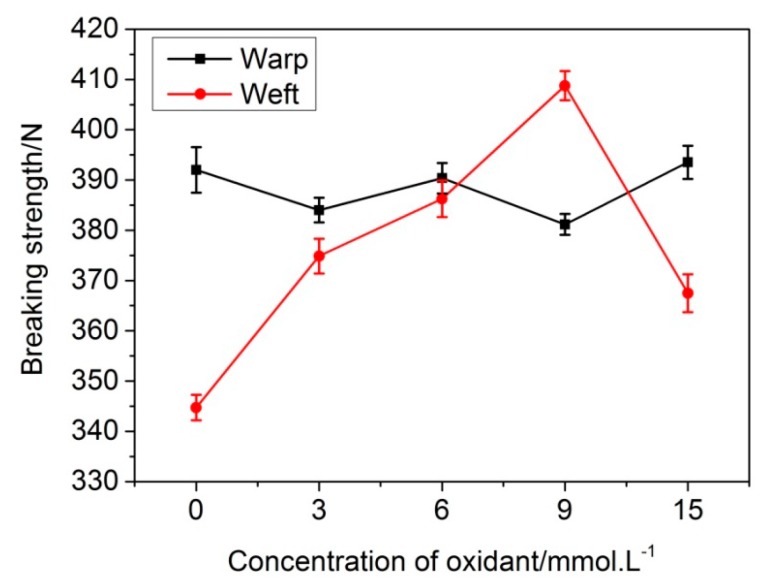
Effect of Ox on the strength of dyed fabric.

The addition of oxidants might damage the surface of silk fibers and affect the strength of dyed fabrics. Consequently, the strength of treated silk fabric was tested. Figure 4 shows the breaking strength of silk fabric under different concentrations of Ox treatment. It can be seen that, with the increase of Ox concentration, the weft strength of silk fabric first increased and then decreased, and the strength reached the maximum value at 9 mmol·L^−1^, which was nearly 15% increased than that without Ox addition. Interestingly, the warp strength was almost unchanged. This phenomenon may be due to the fact that the weft fibers of untreated silk fabric are of low strength and multi-hairiness. The addition of Fe^3+^–Ox led to more DA polymer onto the fabric surface, and the exposed fibers hairiness adhered to the fibers by DA. However, for warp fibers with less hairiness, the effect of the Ox and DA polymerization on the strength of the fabric counteracted, and the warp strength of fabric hardly changed. Moreover, in the process of weaving, the drawing force of the silk fibers in warp directions was larger, and the internal stress was larger. While the weft yarns were less drafted, and the numbers of weft yarns roots in the samples within the same width after DA treatment increased. Accordingly, the weft strength of fabric slightly increased. When the Ox concentration was 9 mmol·L^−1^, the weft strength reached the maximum and the fabric color was the deepest.

### 3.4. Surface Morphology Analysis

**Figure 5:**
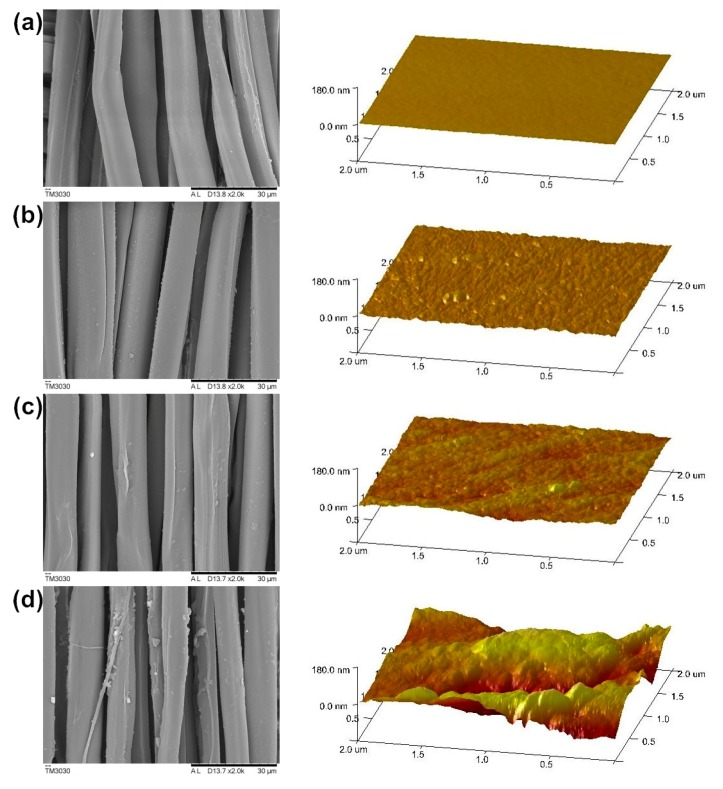

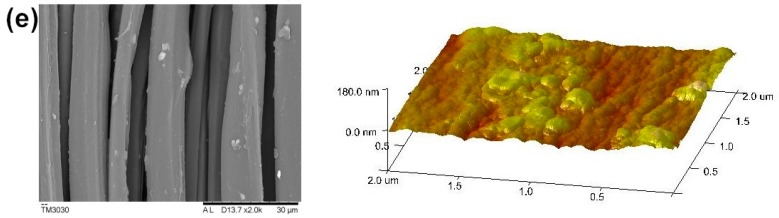
SEM and AFM images about silk samples ((**a**) untreated; (**b**) DA; (**c**) DA–Fe^3+^; (**d**) DA–Ox; (**e**) DA–Fe^3+^–Ox).

The surface morphology of silk fabric before and after DA treatment was observed via scanning electron microscopy (SEM) and atomic mechanical microscopy (AFM) (Figure 5).

The SEM images show that the untreated silk surface was smooth and that the treated silk surface was rough and the granules with different sizes were aggregated on the surface. Figure 5d shows that the silk fibers are damaged greatly after the treatment of the Ox, and the silk fibers are stripped from the silk bundle. However, the silk surface treated by DA–Fe^3+^–Ox is smooth and the fibers are less damaged. This may be because Fe^3+^ covers the surface of fiber and prevents the attack of oxidants on silk fibers. The appropriate addition of Ox accelerates the rate of DA polymerization, and makes the strength of silk stronger.

The AFM image further showed the changes in the surface roughness of the silk fibers before and after DA treatment. Compared with untreated silk fabric, the roughness (Rq) of DA–Fe^3+^- and DA–Fe^3+^–Ox-treated silk fabric increased from 4.20 to 5.90 and 7.77 nm, showing a gradually increasing tendency. It can be expected that the results of this research may provide a theoretical basis for the preparation of hydrophobic fabrics.

### 3.5. XPS Analysis

**Figure 6:**
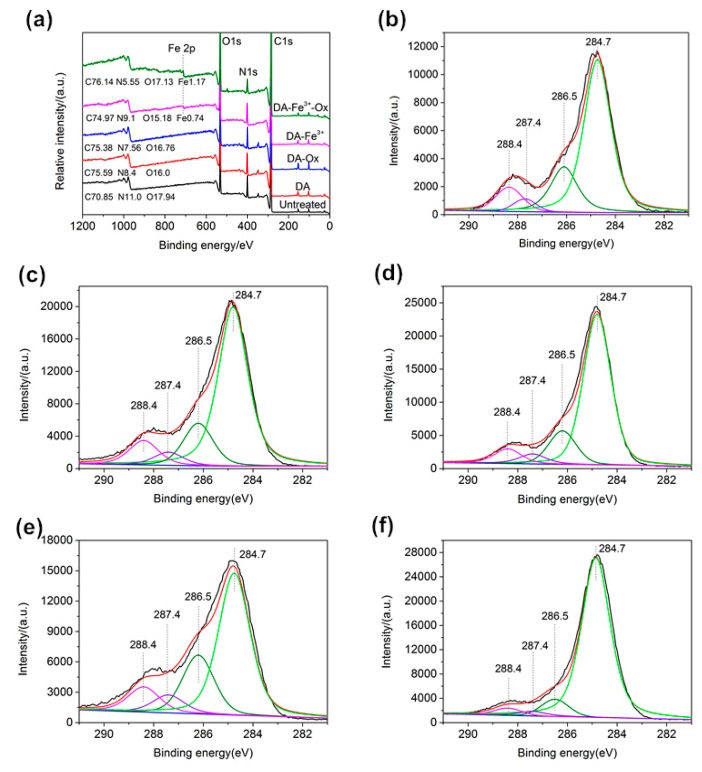

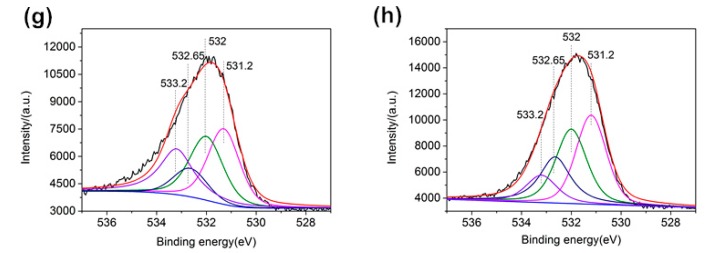
XPS spectra of wide scan spectra (**a**), C1s ((**b**) untreated, (**c**) DA, (**d**) DA–Ox, (**e**) DA–Fe^3+^ and (**f**) DA–Fe^3+^–Ox), and O1s ((**g**) DA–Fe^3+^ and (**h**) DA–Fe^3+^–Ox).

The XPS spectra of untreated silk and DA-treated silk samples were measured. Their wide-angle scanning spectra (a), C1s (b, c, d, e, f), and O1s (g, h) spectra are shown in Figure 6.

From Figure 6a, the relative C content of untreated silk sample and silk samples treated with DA, DA–Ox, DA–Fe^3+^, and DA–Fe^3+^–Ox were 70.85%, 75.59%, 75.38%, 74.97%, and 76.14%, respectively; the relative O content was 17.94%, 16%, 16.76%, 15.18%, and 17.13%, respectively; the relative N content was 11.0%, 8.4%, 7.56%, 9.1%, and 5.55%, respectively. This is because the content of silk and DA is different in element composition, and the amount of PDA deposited on silk surface is also different. In addition, the Fe content values of silk samples treated with DA–Fe^3+^ and DA–Fe^3+^–Ox were 0.74% and 1.17%. XPS mainly tested the content of the elements on the surface of the sample, so the addition of DA could change the content of the elements composition of silk surface, and the DA was successfully finished on the fabric.

Figure 6b–f show the C1s spectra of samples. The absorption peaks of C–C, C–O, C–N, and C–O/C=O appear at 284.7, 286.5, 287.4, and 288.4 eV, respectively. Compared with untreated silk samples, the content values of C–O/C=O and C–N groups in silk treated with DA–Fe^3+^–Ox decreased significantly. This was because a large amount of PDA deposited on the surface of the silk fibers, which increased the content of the C–C group, whereas the content values of the C–O/C=O and C–N groups of PDA were lower than those of the untreated silk fabric. Figure 6g,h show the O1s spectra of samples. The absorption peaks of Fe–O, C=O, Fe–OH, and C–O appear at 531.5, 532, 532.65, and 533 eV, respectively. Compared with the silk samples treated with DA–Fe^3+^, the content of Fe–O and Fe–OH groups of the silk treated with DA–Fe^3+^–Ox increased, and the content of Fe–OH group increased obviously. This may be due to the fact that the addition of Ox accelerated the polymerization of DA, and Fe^3+^ as the bridge of the complex played a crucial role in the silk dyeing.

### 3.6. Anti-Ultraviolet and Hydrophobic Properties Test

Anti-UV performance is one of the important indicators for evaluating the quality of textiles. Table 3 lists the anti-UV properties of untreated silk fabric and the DA-treated samples. The results showed that all DA-treated samples had lower UVA and UVB transmittance and higher UV protection factor (UPF) in comparison with the untreated silk fabrics, among which the DA–Fe^3+^–Ox-treated sample showed the lowest transmittance and highest UPF. The reason was that the DA–Fe^3+^–Ox-treated sample had more PDA, further proving that DA plays an important role in improvement of anti-UV performance. DA was polymerized onto fabrics and formed a large amount of melanin, which improved the UV resistance of treated silk fabric.

The hydrophobic fabric refers to a water contact angle (CA) >90° and the superhydrophobic fabric refers to a CA >150°. The CA test of untreated fabric and silk treated with dopamine was carried out. The results are listed in Table 3, and the CA of untreated fabric and silk treated by dopamine are all 0°, showing good hydrophilicity. As we all know, there are many hydrophilic groups on the sidechain of silk macromolecules [35]. There is also a large number of hydrophilic hydroxyl groups on the surface of PDA. Accordingly, such groups have no hydrophobic property. CA of the DA–Fe^3+^-treated silk fabric reached 126.5°, indicating that the hydrophobic properties of silk fabric could be obviously improved by the treatment of DA and Fe^3+^. The CA of the fabric treated by DA–Fe^3+^–Ox reached 142.1°, which may be due to the increase of the fiber surface roughness Rq under the joint action of Fe^3+^ and Ox, and the fabric showed better hydrophobicity.

## 4. Conclusions

In this study, DA-dyed silk fabric was successfully prepared with the addition of Fe^3+^ and Ox. The obtained fabric was dark black and had certain anti-ultraviolet and hydrophobic properties. When the Ox was added to the reaction solution at 20 min, the Ox and Fe^3+^ concentration was 9 and 2 mmol·L^−1^, the total reaction time was 50 min, and the *K*/*S* value of the fabric was 11.46. The color fastness of the dyed silk was excellent; the light fastness could reach Level 4. It was interesting that the weft strength of the fabric increased by 15% after the addition of Ox, and the warp strength was almost unchanged. The SEM and AFM results showed that the DA–Fe^3+^–Ox-treated samples attached a certain amount of particles and increased the roughness of the fiber surface. The XPS test proved that the surface element content of silk was changed after DA was introduced and Fe^3+^ played a crucial role as a complex. The treated silk fabric showed good anti-UV property with a UPF >30, a UVA <4%, and an excellent hydrophobic property with a CA of 142.1°. Generally speaking, this simple and environmentally friendly method provides a new idea for textile dyeing and functional finishing, which lays a theoretical foundation for the preparation of functional textiles.

## Figures and Tables

**Table 1:** Color characteristic values of silk samples.

Samples	L*	a*	b*	c*	*h*
Untreated	95.16	−0.26	2.23	2.24	96.65
DA	76.07	0.42	1.5	1.56	74.35
DA–Fe^3+^	73.79	1.04	5.48	5.58	79.23
DA–Ox	64.43	0.58	5.16	5.19	83.59
DA–Fe^3+^–Ox	25.59	1.24	−1.33	1.82	313.09

**Table 2:** Washing, rubbing, and light fastnesses of silk fabric dyed with DA.

Samples	Washing Fastness			Rubbing Fastness		Light Fastness
	Fading	Cotton Staining	Silk Staining	Dry	Wet	
DA	4	4–5	4–5	5	4	4
DA–Fe^3+^	4	4	4–5	4–5	4	3
DA–Ox	5	5	5	5	4	4
DA–Fe^3+^–Ox	4	4	4	4	3–4	4

**Table 3:** UV protection factor (UPF) and water contact angle (CA) of silk fabric samples.

Samples	Transmittance/%		UPF	Water Contact Angle (°)	
	UVA	UVB			
untreated	40.67%	18.77%	3.84	0	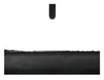
DA	28.31%	13.37%	5.61	0	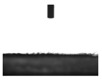
DA–Fe^3+^	22.65%	12.43%	6.38	126.5 ± 1.3	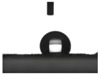
DA–Ox	15.82%	8.62%	9.26	26.7 ± 0.8	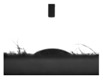
DA–Fe^3+^–Ox	3.73%	2.85%	33.16	142.1 ± 1.5